# A 20-Year Research Trend Analysis of the Influence of Anesthesia on Tumor Prognosis Using Bibliometric Methods

**DOI:** 10.3389/fonc.2021.683232

**Published:** 2021-08-12

**Authors:** Jiamei Luo, Yumiao Shi, Xiaoqiang Wang, Ruirui Zhang, Sifan Chen, Weifeng Yu, Diansan Su, Jie Tian

**Affiliations:** Department of Anesthesiology, Renji Hospital, Shanghai Jiaotong University School of Medicine, Shanghai, China

**Keywords:** anesthetic methods, tumor recurrence, bibliometric analysis, hot spots, CiteSpace

## Abstract

**Background:**

Bibliometric analysis is used to gain a systematic understanding of developments in the field of *the influence of anesthesia on tumor prognosis* and changes in research hot spots over the past 20 years.

**Methods:**

Relevant publications from the Web of Science Core Collection (WoSCC) were downloaded on May 5, 2021. Acquired data were then analyzed using the Online Analysis Platform of Literature Metrology (http://biblimetric.com) and the CiteSpace software was used to analyze and predict trends and hot spots in this field.

**Results:**

1,521 publications on the influence of anesthesia on tumor prognosis were identified and 1494 qualifying records were included in the final analysis. The leading country in this field was the United States of America (USA). The University of Texas MD Anderson Cancer Center (Houston, TX, USA) and Pennsylvania State University (State College, PA, USA) featured the highest number of publications among all institutions. Co-citation cluster labels revealed characteristics of ten main clusters: total intravenous anesthesia, opioid growth factor receptor, gastric cancer cell, opioid receptor, murine model, natural killer cell activity, health-related quality, glioma cell, opioid switching and mu-type opioid receptor. Keyword burst detection indicated that randomized controlled trials (RCTs), volatile anesthetics, and ropivacaine were the newly emerging research hot spots.

**Conclusions:**

This study compiled 1494 publications covering *anesthesia and tumor prognosis* research and showed that the direction of these studies is likely in transition from opioids and their receptors to other anesthetics, and from retrospective studies to prospective randomized controlled trials. It provides guidance for further research and clinical applications on choosing anesthetic methods and drugs.

## Introduction

Cancer has become a major disease threatening the length and quality of people’s lives in modern times. Consequently, Bray et al. predicted that the incidence of all cancer cases may rise to 22.2 million by 2030 (1). Although cancer diagnosis and treatment methods have improved, surgery remains the first-line treatment of solid tumor therapy. However, some researchers raised concerns that surgery-induced stress and inflammatory responses, together with anesthesia, could extend long past the time of surgery, which may affect long-term patient survival (2).

Therefore, an increasing number of researchers have investigated whether anesthetic technique and anesthetics used during cancer resection surgery can influence long-term tumor recurrence or metastasis (2–4). However, research on *anesthesia and cancer* involves many types of cancer and various anesthetic drugs and methods. Thus, it is challenging to grasp a general direction of this body of research and launch investigations in this field if equipped with little or no prior knowledge. While a plethora of experimental and observational clinical data have been published over the past 20 years, systematic summaries of these studies are insufficient. Thus, it is useful to collect data from relevant publications to assist investigators in reading vast amounts of literature on this subject.

Bibliometric analysis is a method used to analyze large amounts of heterogeneous literature; it is based on mathematics and statistics. Combining visualizing processing tools, like CiteSpace, helps gather data on contributions to certain fields from multiple perspectives, including different countries/regions, institutions, journals, authors, co-cited networks, and detailed research trends or hot spots (5).

The aim of this study was to provide a comprehensive understanding of developments in the research field of anesthesia and tumor prognosis by analyzing historic achievements over the past 20 years. Interpreting and summarizing these articles can help predict possible trends and provide a reference for future researchers, especially for those who have an interest, but are new to this field.

## Materials and Methods

### Data Sources and Search Strategies

A literature search was conducted using the Web of Science Core Collection (WoSCC) database on May 5, 2021, to reduce bias incurred by database updating. The search strategy employed was as follows: TI=(an*esthesia or an*esthetic or narcotic or Propofol or etomidate or Opioid or *fentanyl or morphi* or Dexmedetomidine or midazolam or *caine or *flurane or ketamine or naltrexone or naloxone) AND TS=(tum*r or neoplasm or cancer or carcinoma) NOT TS=(non-cancer or “chronic pain”) AND TS=(prognos*s or outcome or recurrence or “overall survival” or “recurrence free survival” or “relapse-free survival” or proliferation or invasion or metastas*s) NOT TI=(guideline or recommendation or consensus or “case report” or meta or review) AND Language=English, and the “Document Type” was set to include “Articles” only from 2001 to 2020. After the primary data search, two researchers (Jiamei Luo and Yumiao Shi) screened all manuscripts individually to ensure they were relevant to the subject of this study.

### Bibliometric Online Platform Analysis

Web of Science (https://wcs.webofknowledge.com) was used to analyze retrieval results and extract the histogram showing the publication trend. For analysis of different countries’ publication trends, the WoSCC data was converted to UTF-8 format and imported into the Online Analysis Platform of Bibliometrics (http://bibliometric.com/) choosing the “total literature analysis” option. For intercountry/regional analysis, we chose the “partnership analysis” option.

### Citespace Software Analysis

Full records and cited references of these publications were downloaded from the WoSCC database and saved as.txt format, and then imported into the Citespace software V5.6R5 SE, 64 bits (Drexel University, Philadelphia, PA, USA), using the following settings: Time slicing from January 2001 to December 2020, years per slice choosing 1. The selection used a modified g-index in each slice: $g2\leq k\;\Sigma_{i\leq g}c_{i},k\in Z^{+},k=25$. For inter-institutional analysis, “Institution” was chosen in the Node Types parameter area, and the rest of the settings were left as default values. For Co-authorship network analysis, “Author” was chosen in the Node Types after importing data into CiteSpace. For document co-citation, the following related parameters were chosen: “References” as the Node Type, “Cosine” to calculate relationship strength, and as the Pruning parameters area “Pathfinder” and “Pruning the merged network” were chosen to simplify the network and highlight its important structural features (6). For keywords burst detection, “Keywords” was chosen as the Node Type. Again, “Cosine” was used to calculate the burst strength. After removing keywords with little real significance (like cells, mice, etc), the top 20 keywords with the strongest burst strength were identified and are displayed in Microsoft Excel 2016.

## Results

### Quantity and Trend Analysis of Published Papers

**Figure 1:**
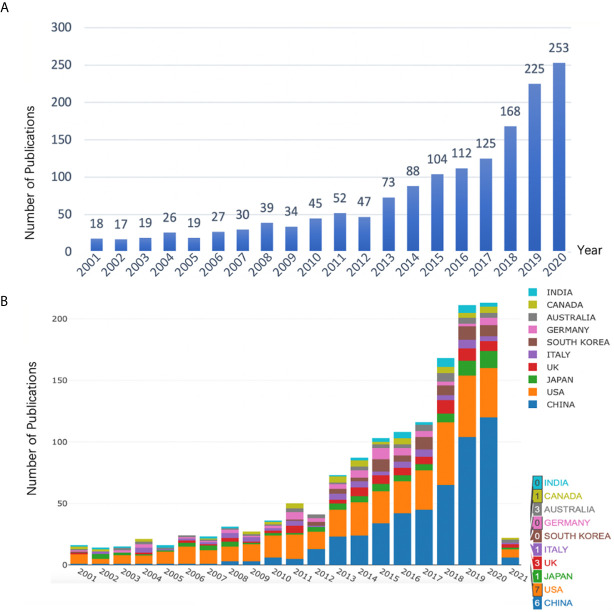
**(A)** Number of annual research publications and growth trends on the topic of anesthesia and tumor prognosis from 2001 to 2020, export of results from Web of Sciences (https://wcs.webofknowledge.com). **(B)** Number of annual publications and growth trends of the top 10 countries/regions on research in anesthesia and tumor prognosis from 2001 to 2021, export of results from the Online Analysis Platform of Literature Metrology (http://biblimetric.com). Bar chart reflects number of online articles online per year.

1521 publications met the inclusion criteria when using our search strategy. After removing duplicate entries, 1482 Articles, 1 Book Chapter, 12 Early Access, 24 Proceedings Papers and 2 Retracted Publications were identified, among which 1494 qualified records (1482 Articles + 12 Early Access) were included in the final analysis. Results showed that research on *anesthesia and cancer* can be roughly divided into two time periods (**Figure 1A**). The early stage (2000-2010) saw fluctuations in the number of publications at a level < 50. However, a trend of increased publications in this field was seen in the 10 years that followed, indicating that the *anesthesia and cancer* field was becoming a research hot spot. Moreover, we used Microsoft Excel 2016 to build a growth trend model as follows: f(x)=ax^3^+bx^2^+cx+d, which indicated that nearly 600 articles will be published by 2025 (**Supplementary Figure 1**).

In order to find out which countries/regions were leading in research in this field, further analysis of publications in different countries and regions was conducted using the Online Analysis Platform of Bibliometrics (http://bibliometric.com/). The bar chart shows the total number of published articles of the top 10 countries/regions over the past 20 years. We found that the United States was a pioneer in this field, and the number of publications has increased steadily. Even though China was initially lagging behind, its annual publication output in this field grew rapidly, outpacing the USA from 2015 onward (**Figure 1B**). Notably, **Figures 1A, B** are from two different websites. **Figure 1A** calculates the number of articles actually published each year, while **Figure 1B** shows the number of articles published online each year. Therefore, **Figure 1B** includes the number of articles published in 2021.

### Analysis of Intercountry/Regional and Inter-Institutional Cooperation

**Figure 2:**
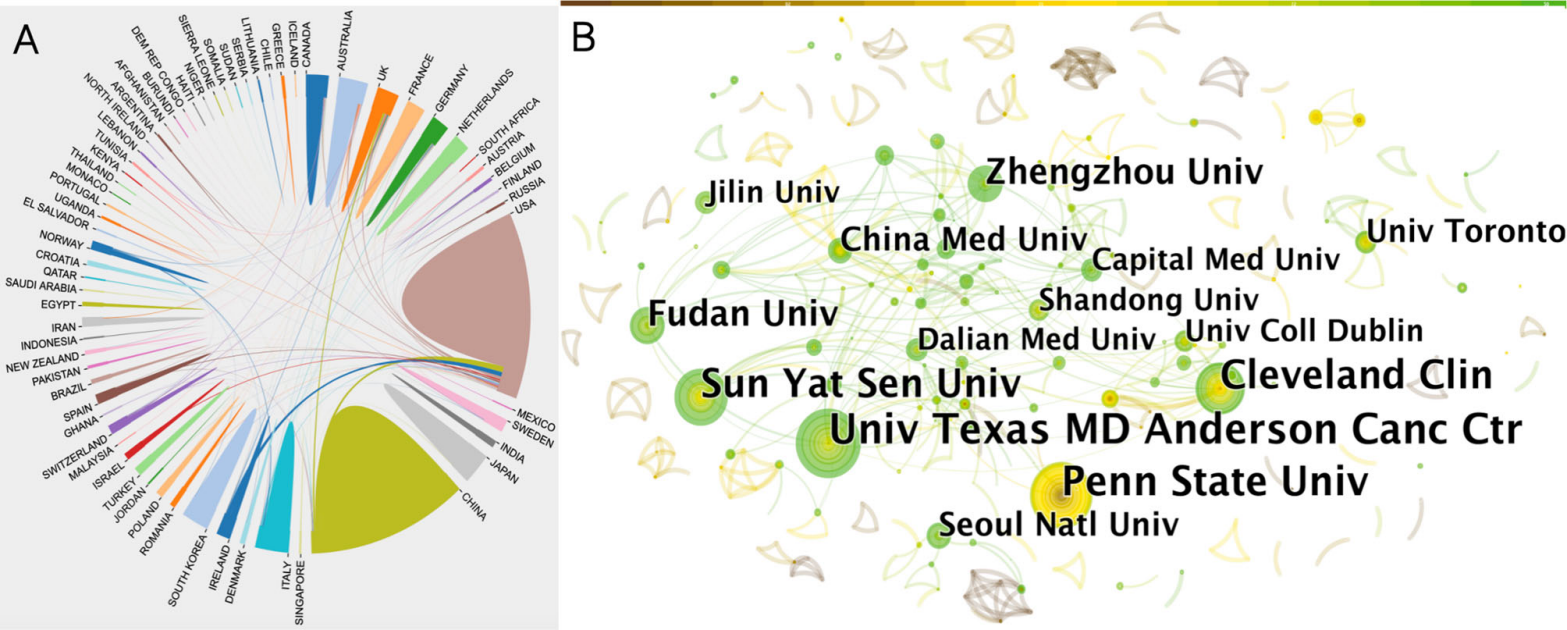
**(A)** Collaboration efforts between 65 countries/regions on the topic of anesthesia and tumor prognosis from 2001 to 2020. Data output from the Online Analysis Platform of Literature Metrology (http://biblimetric.com). **(B)** Citespace network map of institutions involved in anesthesia and tumor prognosis research. Each circle represents an institution. Size of circle is positively correlated with the number of articles published by institutions, and links between two circles represents a collaboration between two institutions on the same article. Line thickness is positively correlated with frequency of collaborations. Top 10 institutions with the most publications are shown. (US Texas MD Anderson Cancer Center, Penn State Univ, Sun-Yat Sen Univ, Cleveland Clin, Fudan Univ, Zhengzhou Univ, China Med Univ, Seoul Natl Univ, Univ Toronto were top 9 and Dalian Medical University, Jilin University, University college Dublin, Shandong University and Capital Medical University tied for tenth). Timespan: 2001-2020; Slice length = 1.

Next, we analyzed cooperation efforts among different countries using the bibliometrics online analysis platform (**Figure 2A**). Results of intercountry/regional cooperation suggested that 65 countries worked in partnerships, especially the USA and China. However, China showed less international cooperation compared to the USA.

In order to find out about research institutional and interinstitutional cooperation efforts in the *anesthesia and cancer* field, we next imported TXT format files into the CiteSpace software. Results of the collaborative relationship between different institutions showed 537 nodes and 523 links (**Figure 2B**). The top two of the most prolific institutions, the University of Texas MD Anderson Cancer Center and Pennsylvania State University, were both located in the USA, followed by a Chinese institution, Sun Yat-sen University, also indicating that contributions from the USA and China cannot be ignored in this field (**Figure 2B**).

The names of the top 10 most productive institutions were labeled. Since five institutions had the same number of publications and shared the tenth place, there are a total of 14 institutions displayed in **Figure 2B**. The size of the concentric circles represents the number of publications, therefore, institutions with more published articles are represented with larger concentric circles. Some institutions are too small to be identified. Links between two institutions represent collaboratively published articles. Line thickness indicates the strength of the cooperation. A network density of only 0.0036 indicated that cooperation between these organizations was not close enough. Interestingly, there was a closer connection between institutions with fewer publications (**Figure 2B**).

### Co-Authorship Network and Core Author Distribution Analysis

**Figure 3:**
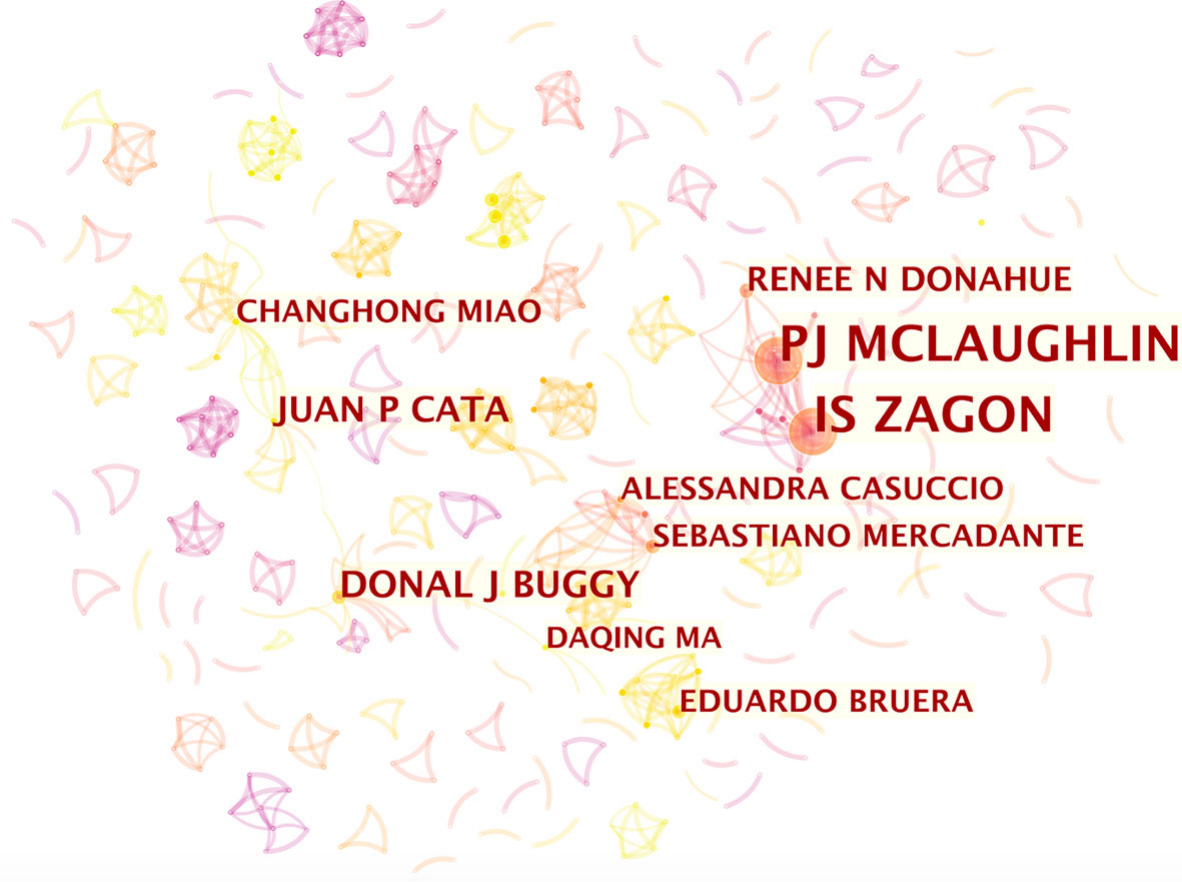
Citespace network of co-authorship in the field of anesthesia and cancer research. Each circle represents one author. Size of circle is positively correlated with the number of articles published by authors, and links between two circles represents a collaboration between two authors on the same article. Line thickness is positively correlated with frequency of collaborations. Top 10 authors with the most publications are shown. Timespan: 2001-2020; Slice length = 1.

In most cases, multiple researchers are required to collaborate on a study, and their contributions are represented as a ranking of authors. We can evaluate the core authors and their cooperation in a certain field by analyzing the characteristics of authors’ cooperative networks. Results include 669 nodes and 722 links. The top 10 researchers and their teams in this research area are shown in **Figure 3**. Font size is positively associated with numbers of the authors’ publication. IS Zagon (39 articles) and Patricia J Mclaughlin (39 articles), both from Pennsylvania State University, were first and second on the list, respectively (**Figure 3**). The other eight major research teams are also displayed in **Figure 3** (Juan P Cata from the University of Texas and MD Anderson Cancer Center, Donal J Buggy from Mater Misericordia University Hospital, Renee N Donahue from National Cancer Institute, Bethesda Maryland USA, Sebastiano Mercadante from La Maddalena Cancer Center, Palermo, Italy, Alessandra Casuccio from University of Palermo, Italy, Daqing Ma from Chelsea & Westminster Hospital, Eduardo Bruera from University of Edinburgh, Royal Infirmary and Changhong Miao from Zhongshan Hospital, Fudan University). Contrary to the results of the institution analysis, authors who published more articles tended to collaborate more closely with others.

### Journal Analysis

The WoSCC showed that the 1,494 papers included in the current analysis were published in 604 different journals over the past 20 years (2001-2020). Bibliometrics online analysis platform was used to analyze journal influence. The top 10 most cited journals are listed in **Supplementary Table 1**. Four publishers of these 10 most cited journals are in the United States (USA), including the highest-ranking journal *Anesthesiology*; two are in the United Kingdom (UK); and the other four are in Greece, Ireland, France and Italy, respectively. *Anesthesiology*, which showed the highest number of total citations (628) with an IF of 7.892, ranked first in the research field of the influence of anesthesia on tumor prognosis.

### Document Co-Citation and Clustered Network Analysis

Document co-citation is a method developed by bibliometric research to identify literature co-cited by a group of authors. In other words, this analysis is used to measure the relationship of two documents by visualizing their co-occurrence of citations (7). Citespace software was used to analyze 1494 original articles and their 36072 valid references to identify distinct homogenous clusters of highly cited documents in the anesthesia and tumor research field. The 36072 references included reviews and other *secondary literature*.

**Figure 4:**
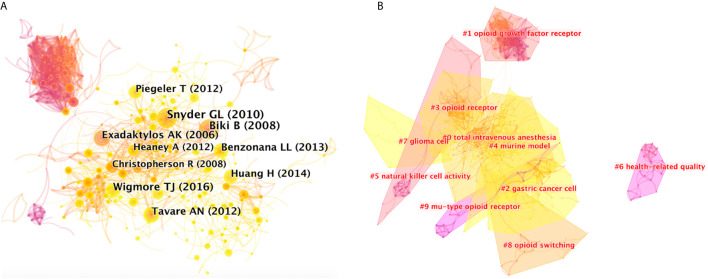
**(A)** Citespce co-citation map of 36072 references on anesthesia and cancer research, filter option showing the largest connected component only. Each circle represents a reference. Size of circle is positively correlated with frequency of citations, and links between two circles represent two references that were cited in the same article. Year and first author of the top 10 most cited publications are shown. Timespan: 2001-2020; Slice length = 1. **(B)** Clustered networks of co-citation status of the investigated 36072 references and the 1494 citing articles *via* CiteSpace. The top 10 largest clusters of citing articles are shown.

**Figure 4A** shows co-citations of the 36072 references, and the year and the first author of the top 10 most cited references. Each circle represents a reference. Circle size is positively correlated with the frequency of citations, and links between two circles represent two references that were cited within the same article among the retrieved 1494 articles (citing articles) of the present study. Similarly, line thickness is positively correlated with the frequency of co-citations. Details of the top 10 most cited references are listed in **Supplementary Table 2**.

Results showed that the highest-ranking reference was a review published by *the British Journal of Anesthesia* in 2010 (8). It suggested that anesthetic technique and medication choice can interact with the cellular immune system and affect long-term outcomes. Results of limited human studies indicate that regional anesthesia may be beneficial, which is consistent with the results of the article with the second highest citation, a retrospective analysis published by *Anesthesiology* in 2008 (9). Researchers evaluated 225 patients with invasive prostatic carcinoma, and results showed that general anesthesia plus epidural analgesia, instead of general anesthesia plus opioid analgesia, has less risk of biochemical cancer recurrence. The third highest-ranking article was another retrospective study published by *Anesthesiology* in 2016 (10), which analyzed 7030 patients undergoing elective surgery due to solid tumors. It concluded that total intravenous anesthesia (TIVA), compared with volatile inhalational (INHA), can improve long-term survival in patients presenting for elective surgery in a comprehensive cancer center over 3 years. Since literature is usually cited to support the opinions of authors, high citation would generally reflect that these references contain concept symbols which have received peer recognition, and it is an indication that they have made important past contributions in the field.

Clustered networks were then generated in a hierarchical order, based on the co-citation status of the 36072 references by the 1494 citing articles *via* CiteSpace, because if two publications have many references in common, they tend to be homogenous. The ten major clusters of the 1494 citing articles are shown in **Figure 4B**. Cluster labels were salient noun phrases extracted from titles of the citing articles using the LSR algorithm, including #0 total intravenous anesthesia, #1 opioid growth factor receptor, #2 gastric cancer cell, #3 opioid receptor, #4 murine model, #5 natural killer cell activity, #6 health-related quality, #7 glioma cell, #8 opioid switching, #9 mu-type opioid receptor (**Figure 4B**). The number of cluster tags are reversely correlated with the number of articles for each cluster included. In other words, the cluster of #0 contains the largest number of articles. A summary of clusters is listed in **Supplementary Table 3**.

### Research Trend Analysis and Burst Detection With Keywords

**Figure 5:**
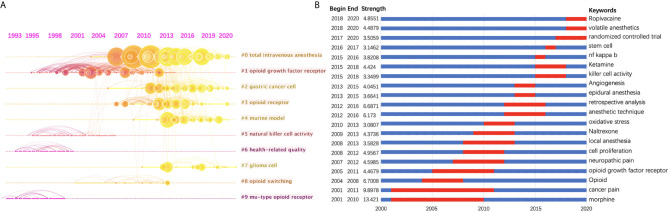
**(A)** Timeline view of the top 10 largest clusters of citing articles in the field of anesthesia and cancer research. Right side = cluster labels. **(B)** Keywords with the strongest burst strength of the 1494 citing articles on anesthesia and cancer research between 2001 and 2020. Keywords marked in red indicates a sudden increase in usage frequency of this keyword during that period. Blue represents a relatively unpopular time period.

In order to show the clusters of the citing articles more clearly, a timeline view of these clusters is shown in **Figure 5A**. A bolded timeline in **Figure 5A** indicates that the clustering topic was a hot spot during this period of time. Citation tree-rings of different sizes on the timeline represent key references with high citation rates. We found that in the field of *anesthesia and tumor*, mu-type opioid receptor has been a hot topic since 1993 until the end of 20^th^ century. It has become a research focus again since around 2006, together with other types of opioid receptors, i.e., kappa-type, delta-type, etc. Total intravenous anesthesia has also attracted increasing attention in recent years. Furthermore, according to the clustering results, gastric cancer and glioma are two kinds of tumors that many people are interested in in recent studies. Natural killer cell activity may be the mechanism underlying the influence of anesthetics on tumor progression.

Keyword burst detection was another method to show research trends (**Figure 5B**). A red line indicates that the use of this keyword increased suddenly during this period. In contrast, a blue line represents relative unpopularity. Morphine, with a strength of 13.421, was ranked first, followed by cancer pain (9.8978), which were considered a research focus during the years from 2001 to 2010 and 2011. The impact that anesthesia methods (local anesthesia, anesthetic technique, epidural anesthesia, etc.) and drugs (such as volatile anesthetics, ropivacaine, etc.) exerted on cancer recurrence has drawn much more attention since 2008. In addition, we found that randomized controlled trials (RCTs) have become a research focus from 2017 onwards.

## Discussion

This current study visualized research articles in the field of *influence of anesthesia on tumor prognosis* from 2001 to 2020. The number of published articles on this topic rapidly increased after 2010 and reached almost 200 articles per year by 2020. However, the actual research topics were relatively diverse. Using an online bibliometric analysis platform and CiteSpace software, our study analyzed publications about *anesthesia and cancer* research from multiple dimensions, and showed a systematic view in understanding in this field over the past 20 years and provided guidance for future studies. Researchers new to this field of study now can easily get useful and relevant information with the help of our bibliometric analysis.

The top 3 countries that focus on the field of *influence of anesthesia on tumor prognosis* are the USA, China, and Japan. Notably, by 2015 the number of articles from China surpassed those from the USA, and China became the most prolific country of origin of publications in this field. However, China dropped out of the top 10 when quantifying the significance of their contributions compared to other countries. This suggests that Chinese researchers are quite interested in this topic, however, quality and influence of their research still needs improvement.

International cooperation, especially between the USA and China, was common. The top two most prolific research institutions were both located in the USA, followed by Sun Yat-sen University from China. Of the 604 different journals that published 1,521 papers in this field, 40% are from USA, making the USA the leading country in the field.

The timeline view of the 36072 related references and keyword burst detection both indicate trends in the field of *anesthesia and cancer* research. The effects of opioids, especially morphine, on tumor progression has attracted the researchers’ attention since the beginning and lasted for decades (11–14). The effects of other anesthetics, including local anesthetics and volatile anesthetics, have also become a research interest in recent years.

At present, it is still a matter of debate how these different anesthesia methods and anesthetics influence the prognosis for patients undergoing tumor resection, but there is enough current evidence to generate hypotheses that they may affect long-term oncologic and survival outcomes. The current bibliometric analysis indicated that a plethora of retrospective clinical and experimental data has been published over the past 20 years, and most researchers showed that regional anesthesia is more favorable for a good prognosis for surgical oncology patients compared to general anesthesia (9, 15–23). Research also suggested that intravenous hypnotic drugs [like propofol (24–26)] and local anesthetics [like lidocaine (27) and ropivacaine (28)] provide an anti-cancer effect, whereas opioids (11) and volatile anesthetics (29, 30) may promote tumor development. However, the available evidence was not strong enough to change clinical practice. Consequently, as shown in the keyword burst detection results of this study, more RCTs are conducted by researchers in recent years in this research field to seek clinical evidence. Hopefully, they can address this important clinical question in an effective way.

Further work is required to fully understand the mechanisms driving the above mentioned phenomena. Consequently, this study found that immune response, such as the activity of natural killer cells, has been a research hot spot (31). It is now widely accepted that circulating immune cells in human blood can recognize tumor-associated antigens. This immunosurveillance protects the host against cancer development, and immunosuppressants are often associated with high incidences of cancer (32). Many preclinical and clinical studies found that anesthetic drugs can directly or indirectly modulate the immune system (33). Melamed et al. reported that ketamine, halothane, and thiopental can suppress natural killer cell activity and promote tumor metastasis. Conversely, propofol does not have these effects (34). In addition, opioids, widely used during anesthesia and perioperative period, were also proved to reduce activation of the pro-inflammatory transcription factor NF-κB, which was also detected by the Keywords burst detection in this study, and affect the adaptive immune system (33, 35). Recent studies showed that morphine promotes the migration of MCF-7 cells *via* the TLR4/NF-κB signaling pathway (36).

Anesthesia methods and drugs can also influence tumor progression by influencing the malignant biological behavior of tumor cells. The effect of opioids facilitating cancer cell proliferation was proven in both cell culture experiments and animal models (3, 37–39). Similar findings were reported when using volatile anesthetics (40, 41). In contrast, propofol and local anesthetics, i.e., lidocaine, have been shown to inhibit cell proliferation and migration in different kinds of cancer cells (24, 26, 27, 42, 43).

The current study has several limitations. First, the current analysis is based on citation data. Reviews and articles are two document types frequently cited, whereas very little citation data is available for books or conference papers. Therefore, some of these publications are not tracked by bibliometric searches. Second, only articles with English keywords or abstracts in the WOS database were considered in our analysis due to requirements by the CiteSpace software. High-quality articles in other languages, although few, were not cited. Third, bibliometrics is a quantitative analysis of scholarly publications, where high citation counts may not necessarily indicate quality. For example, a highly-cited article does not necessarily correspond to clinical demands, and sometimes it might even not be clinically relevant, especially for basic research articles focusing on a single molecule or a single pathway. In the future, we may use multimethod evaluations to gain a more in-depth understanding of this research field.

In conclusion, bibliometric analysis offers an objective and quantitative method for assessing publication performance between countries, researchers, research institutions, etc. Our results showed considerable interest in the field of *anesthesia and tumor prognosis* research in recent years, particularly the study of opioids and their receptors, local anesthetics and volatile anesthetics. Moreover, the keyword burst detection in this study also indicated that different anesthesia techniques and anesthetics have different effects on the prognosis of cancer patients, which would necessitate more RCTs. Their guidance for clinical practice may benefit countless patients with cancer.

## Author Contributions

JL and YS: These two authors contributed equally to this work helping raising the conception of the study, searching and screening articles, processing data of the study, drafting and reviewing the manuscript. XW, RZ, and SC: These authors contributed to the conception of the study, reviewed and edited the manuscript. WY, DS, and JT: These authors helped to conceive the idea of the study, critically review and edit the manuscript. All authors contributed to the article and approved the submitted version.

## Funding

This study was supported by grants from Shanghai Shenkang Hospital Development Center Three-year Funding for Major Clinical Research Projects (SHDC2020CR4062), Natural Science Foundation of Shanghai Science and Technology Committee (19ZR1430600) and Shanghai Municipal Key Clinical Specialty (shslczdzk03601).

## Conflict of Interest

The authors declare that the research was conducted in the absence of any commercial or financial relationships that could be construed as a potential conflict of interest.

## Publisher’s Note

All claims expressed in this article are solely those of the authors and do not necessarily represent those of their affiliated organizations, or those of the publisher, the editors and the reviewers. Any product that may be evaluated in this article, or claim that may be made by its manufacturer, is not guaranteed or endorsed by the publisher.

## Supplementary Material

Supplementary Figure 1 Output of publications and growth prediction of anesthesia and cancer research. The number of publications from 2001 to 2020 are represented by large scatters; the dashed line represents the predicted curve, R^2^ = 0.9877.

## Abbreviation

WoSCC, Web of Science Core Collection; USA, United States of America; PA, Pennsylvania; TX, Texas; OGF, Opioid growth factor; RCTs, Random control trials; TI, Title; TS, Topical subject; IF, Impact factors.
