# Supplementary figures and images for: A 20-Year Research Trend Analysis of the Influence of Anesthesia on Tumor Prognosis Using Bibliometric Methods
Source: Front Oncol. 2021 Aug 12;11:683232. doi: 10.3389/fonc.2021.683232 (PMC8397496; doi:10.3389/fonc.2021.683232)

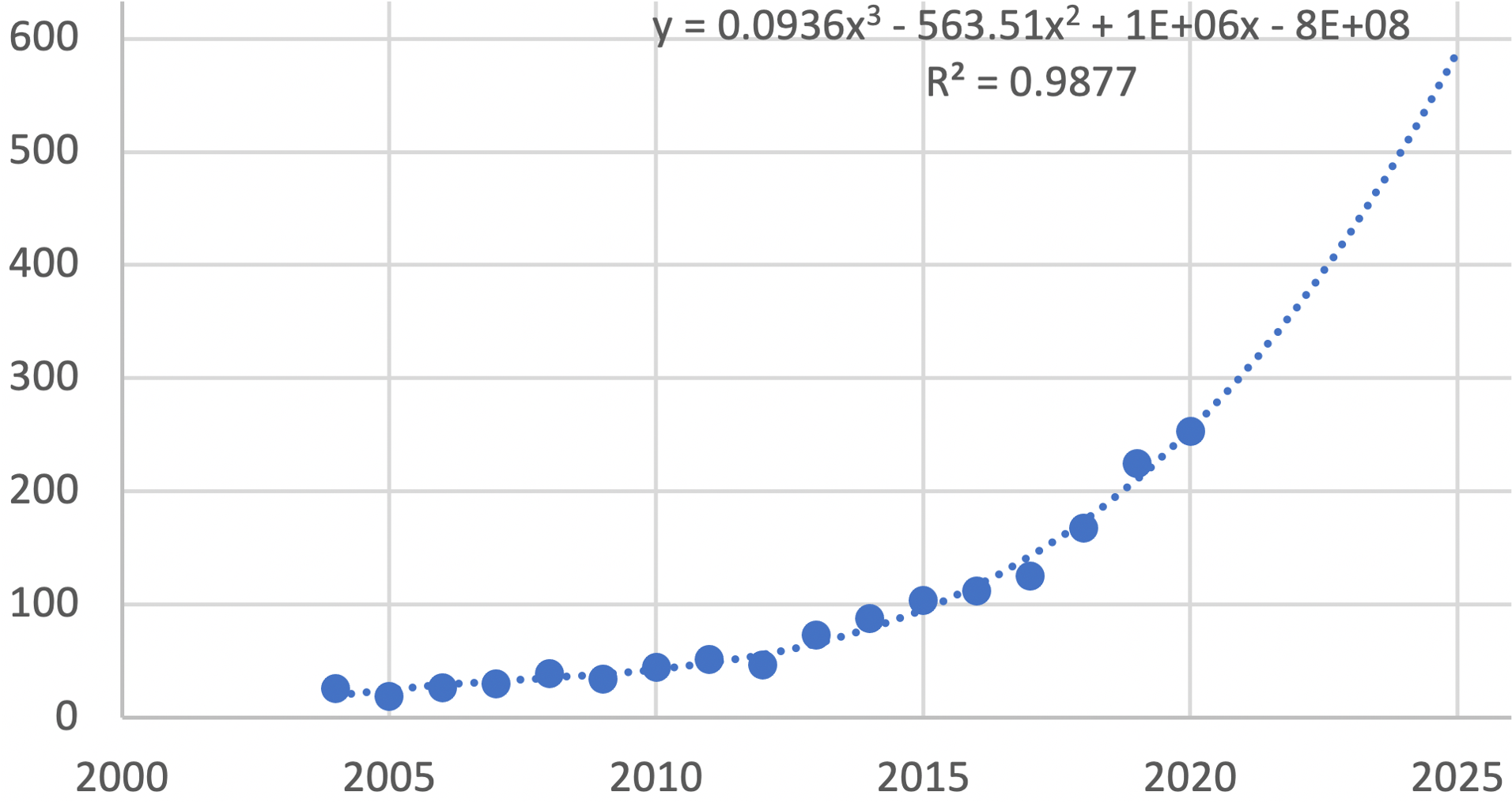

Supplement: Supplementary Figure 1 — Output of publications and growth prediction of anesthesia and cancer research. The number of publications from 2001 to 2020 are represented by large scatters; the dashed line represents the predicted curve, R2 = 0.9877. [file Image_1.tif]
