# Supplementary material for: A 20-Year Research Trend Analysis of the Influence of Anesthesia on Tumor Prognosis Using Bibliometric Methods
Source: Front Oncol. 2021 Aug 12;11:683232. doi: 10.3389/fonc.2021.683232 (PMC8397496; doi:10.3389/fonc.2021.683232)
Supplement: Supplementary file 2 [file Table_1.docx]

**Supplementary**

**Supplemental Table 1. The top 10 most active journals that published articles in anesthesia and cancer research (sorted by total citation)**

| Rank | Journal Title | Frequency | Total citations | Average citation per paper | Impact factor  (2019) | Country | JCR |
| --- | --- | --- | --- | --- | --- | --- | --- |
| 1 | Anesthesiology | 26 | 628 | 24.15 | 7.067 | USA | / |
| 2 | British Journal of Anesthesia | 47 | 511 | 10.87 | 6.88 | UK | Q1 |
| 3 | Anesthesia and Analgesia | 27 | 308 | 11.41 | 4.305 | USA | / |
| 4 | Cancer Research | 3 | 139 | 46.33 | 9.727 | USA | Q1 |
| 5 | Anticancer Research | 17 | 137 | 8.06 | 1.994 | Greece | Q3 |
| 6 | Cancer Letters | 6 | 109 | 18.17 | 7.36 | Ireland | Q1 |
| 7 | Plos One | 22 | 103 | 4.68 | 2.74 | USA | Q1 |
| 8 | Biomedicine& Pharmacotherapy | 10 | 102 | 10.20 | 4.545 | France | / |
| 9 | European Review for Medical and Pharmacological Sciences | 18 | 84 | 4.67 | 3.024 | Italy | / |
| 10 | British Journal of Cancer | 3 | 84 | 10.20 | 5.791 | UK | Q2 |

**Supplemental Table 2. The top 10 high-cited papers in anesthesia and cancer research during 2001 to 2020**

| Rank | Title | First Author | Journal | Year | Cited Frequency | DOI |
| --- | --- | --- | --- | --- | --- | --- |
| 1 | Effect of anaesthetic technique and other perioperative factors on cancer recurrence | Snyder GL | BRIT J ANAESTH | 2010 | 80 | 10.1093/bja/aeq164 |
| 2 | Anesthetic Technique for Radical Prostatectomy Surgery Affects Cancer Recurrence | Biki B | ANESTHESIOLOGY | 2008 | 74 | 10.1097/ALN.0b013e31817f5b73 |
| 3 | Long-term Survival for Patients Undergoing Volatile versus IV Anesthesia for Cancer Surgery: A Retrospective Analysis. | Wigmore TJ | ANESTHESIOLOGY | 2016 | 60 | 10.1097/ALN.0000000000000936 |
| 4 | Cancer recurrence after surgery: direct and indirect effects of anesthetic agents | Tavare AN | INT J CANCER | 2012 | 55 | 10.1002/ijc.26448 |
| 5 | Isoflurane, a commonly used volatile anesthetic, enhances renal cancer growth and malignant potential via the hypoxia-inducible factor cellular signaling pathway in vitro. | Benzonana LL | ANESTHESIOLOGY | 2013 | 52 | 10.1097/ALN.0b013e31829e47fd |
| 6 | Can anesthetic technique for primary breast cancer surgery affect recurrence or metastasis? | Exadaktylos AK | ANESTHESIOLOGY | 2006 | 52 | 10.1097/00000542-200610000-00008 |
| 7 | Prostate cancer cell malignancy via modulation of HIF-1α pathway with isoflurane and propofol alone and in combination. | Huang H | Br J Cancer | 2014 | 47 | 10.1038/bjc.2014.426 |
| 8 | Antimetastatic Potential of Amide-linked Local Anesthetics Inhibition of Lung Adenocarcinoma Cell Migration and Inflammatory Src Signaling Independent of Sodium Channel Blockade | Piegeler T | ANESTHESIOLOGY | 2012 | 45 | 10.1097/ALN.0b013e3182661977 |
| 9 | Can anaesthetic and analgesic techniques affect cancer recurrence or metastasis? | Heaney A | BRIT J ANAESTH | 2012 | 42 | 10.1093/bja/aes421 |
| 10 | Long-Term Survival After Colon Cancer Surgery:A Variation Associated with Choice of Anesthesia | Christopherson R | ANESTH ANALG | 2008 | 38 | 10.1213/ane.0b013e3181770f55 |

**Supplemental Table 3. Summary of 10 Clusters.**

| **Cluster ID** | **Top term** | **Size** | **Silhouette** |
| --- | --- | --- | --- |
| 0 | total intravenous anesthesia | 132 | 0.887 |
| 1 | opioid growth factor receptor | 81 | 0.967 |
| 2 | gastric cancer cell | 64 | 0.967 |
| 3 | opioid receptor | 62 | 0.944 |
| 4 | murine model | 50 | 0.898 |
| 5 | natural killer cell activity | 22 | 0.987 |
| 6 | health-related quality | 20 | 0.982 |
| 7 | glioma cell | 20 | 1 |
| 8 | opioid switching | 18 | 0,988 |
| 9 | mu-type opioid receptor | 17 | 0.911 |

Silhouette value >0.5 means the clustering results are reliable.
